# Carbapenem-resistant *E. coli* with one-health approach in East Africa: Systematic review and meta-analysis

**DOI:** 10.1016/j.puhip.2026.100843

**Published:** 2026-08-22

**Authors:** Tizazu Zelelie, Biniam Mekonnen, Demiss Nigussie

**Affiliations:** a Department of Medical Laboratory Science, Debre Berhan University, Asrat Woldeyes Health Science Campus, Debre Berhan, Ethiopia; b Department of Paediatrics and Child Health, Debre Berhan University, Asrat Woldeyes Health Science Campus, Debre Berhan, Ethiopia

**Keywords:** Carbapenem-resistance, *E. coli*, One-health, East African

## Abstract

**Objective:**

Carbapenem-resistant *E. coli* is one of the most serious and urgent global health threats. This study aimed to explore the occurrence and characteristics of carbapenem-resistant *E. coli* in East African countries using a one-health approach.

**Study design:**

A systematic review and meta-analysis study.

**Methods:**

This review was done based on the Preferred Reporting Items for Systematic Reviews and Meta-Analyses (PRISMA) reporting guidelines. The protocol of this study was also registered on the International Prospective Register of Systematic Reviews (PROSPERO) database (Registration Identification: CRD42024626968).

**Results:**

The pooled prevalence of carbapenem-resistant *E. coli* in East Africa among different interfaces like human, animal, environment, and food was 12.7% (95% CI: 8.6, 16.8). The environment exhibited the predominant interface for carbapenem-resistant *E. coli* (24.2%), followed by human (11.9%), food (7.7%) and animals (4.2%). The carbapenem-resistant *E. coli* prevalence decreased in 2021 - 2025 (12.6%) as compared to 2015-2020 (13.5%). High prevalence was seen in Sudan (24.7%), Uganda (17.4%), and Ethiopia (9.9%) and predominantly found in environmental samples (24.2%) and various clinical samples (15.8%). Different resistance gene variants, including serine β-lactamases, metallo-β-lactamases, and oxacillinase, were found in 51.7% of the studies.

**Conclusions:**

Carbapenem-resistant *E. coli* is prevalent and poses a potential future threat in East African countries. It needs multifaceted strategies, including regional integration beyond health policy to mitigate the problem.

## 1 Introduction

From the East African countries, some of them are categorized as middle-income countries and others, except Seychelles, are low-income countries. East Africa is the only region in Africa that avoided a recession in Gross Domestic Product (GDP) in 2020. [1] The economic activities in the region include agriculture and pastoralism that could result in improper use of antimicrobial drugs practices. [2] In addition, lack of access to clean and safe water, poor sanitation and hygienic practice, and inadequate infection prevention measures are challenges in the region. [3] This could contribute to the emergence and spread of antimicrobial resistance (AMR). A one-health approach for understanding the problems could be an appropriate strategy. The approach has been internationally endorsed in 2015 by the tripartite organizations (the Food and Agriculture Organization, the World Health Organization [WHO], and the World Organization for Animal Health), later by Quadripartite Organizations (in addition to the tripartite organizations, the United Nations Environment Programme joined them) to address risks at the human–animal–environment interface. [4] In line with this, the African Union Regional Quadripartite has been formed for similar purposes. [3] Resistant bacteria and their genes can reach the environment and by horizontal gene transfer could spread to other human and animal hosts. [5]

Currently, AMR bacterial infections linked to one in eight of the global deaths [6] and become a global health threats. [6,7] There were nearly 5 million deaths associated with AMR bacterial in 2021 globally. [6] About 8.22 million deaths associated with AMR could occur globally in 2050 if the problem continued with the current trends. [6] In 2019, there were 3.8 million deaths globally. Of these, nearly 2 million deaths were caused by bacterial infection and more than one million deaths associated with AMR bacteria in the WHO African region. [7] In East African countries, AMR including carbapenem-resistant could be more severe due to the presence of many driving factors. [8] Some of the factors include a dearth clinical microbiology service, and poor geographic access to healthcare services.

Carbapenems are drugs of beta-lactams groups and most effective against Gram positive and Gram negative bacteria. [9] They are prescribed for multi-drug resistant (MDR) bacterial infections and considered as last-resort treatment options. Bacteria become resistant to carbapenems due to genetic changes, diminished expression or loss of target, expression of efflux pump, and production of beta-lactamases. [9] Carbapenem-resistant Enterobacteriaceae (CRE) infections have been identified as one of the causes for longer lengths of stay, increased health care costs, and higher mortality. [5,9] Among Gram-negative bacteria, resistance to carbapenems increased more than any other antibiotic class, cause deaths rising from 619,000 in 1990 to 1.03 million in 2021. [6] According to the WHO global priority list of antibiotic resistant bacteria, carbapenem-resistant is categorized as number 1 pathogens, helps in prioritizing the urgent to research and development of new and effective antibiotic treatments. [10] There is very limited treatment options for carbapenem resistant Gram-negative pathogens (polymyxins, fosfomycin and tigecycline) compared to carbapenem resistant Gram positive pathogens. [9]

*E. coli* strains are global targets for AMR research [11,12] and used as indicator organisms in different surveillance programs [12,13] including carbapenems-producing *E. coli* strains. Different surveillance programs are established for pathogenic *E. coli* to monitor and track outbreaks in developed countries including in United Kingdom [14] and America. [15] Limited comprehensive data on carbapenem-resistant *E. coli* hinders the development of better preventive and treatment strategies at the regional level. The present study aimed to determine the epidemiology of Carbapenem-resistant *E. coli* with one health approach including in human, animal, and environmental interfaces in East Africa.

## 2 Methods

### 2.1 Study design

The study design is systematic review and meta-analysis. The study was done based on the Preferred Reporting Items for Systematic Reviews and Meta-Analyses (PRISMA) reporting guidelines. [16] The protocol of this study was registered on the International Prospective Register of Systematic Reviews (PROSPERO) database (Registration Identification: CRD42024626968).

### 2.2 Literature search strategy and selection

**Fig. 1:**
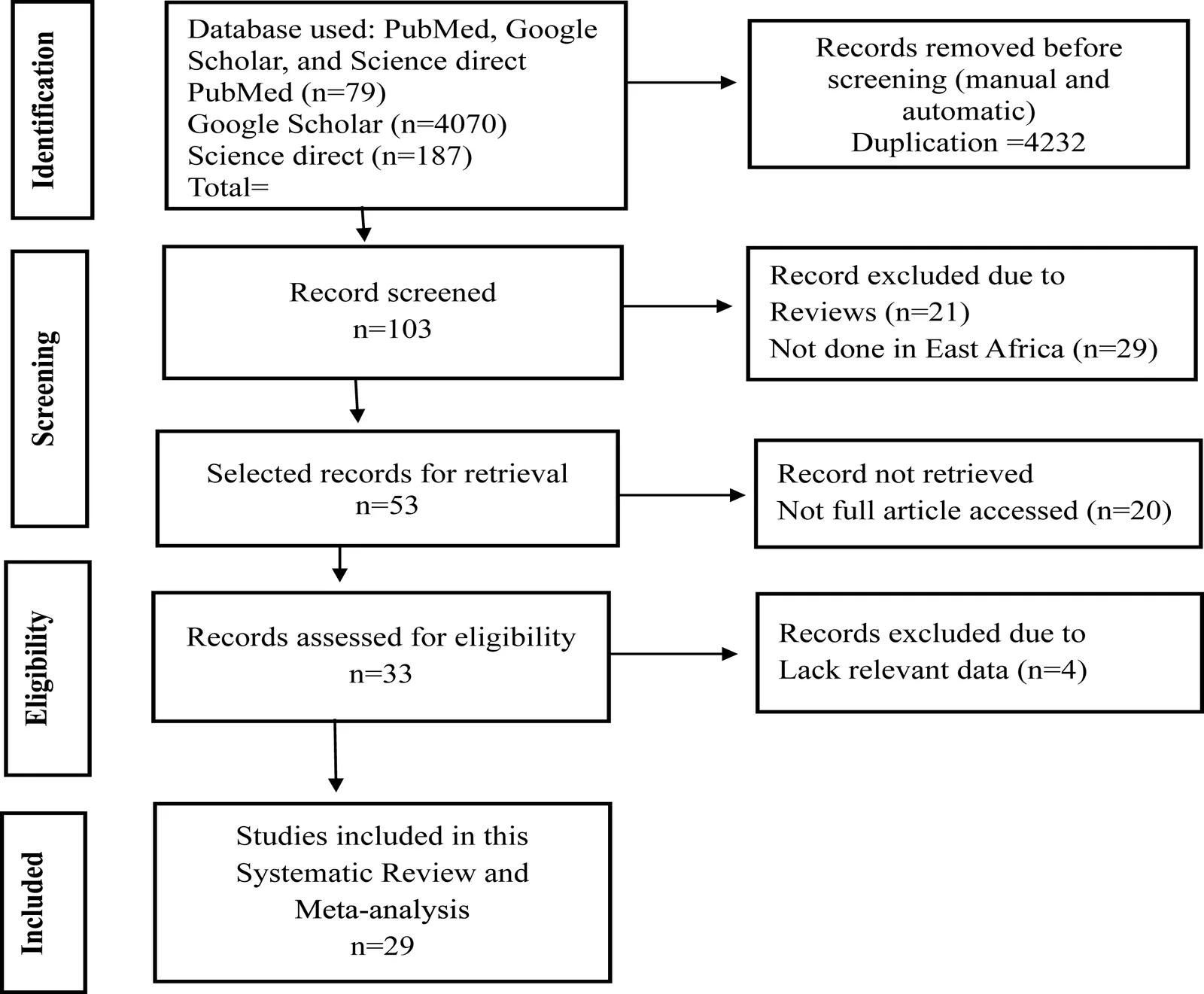
Schematic representation of the search strategy for records and articles using the PRISMA criteria. [16]

A comprehensive literature search was conducted to find selected studies on the prevalence of carbapenem-resistant *E. coli* in East Africa that were available in the PubMed, Science Direct, and Google scholar (Fig. 1). The process includes downloading journal articles from these databases, screening by their title and abstract, and data extraction. The extracted data was then exported in the comma-separated values (CSV) file format for further analysis in Microsoft Excel, and R Studio. Terms and keywords were used to retrieve all relevant articles from the three databases. The main search terms were “Carbapenem-resistant”, “Carbapenemase producing”, “*Escherichia coli*”, “*E. coli”,* Burundi, Comoros, Djibouti, Eritrea, Ethiopia, Kenya, Rwanda, Seychelles, Somalia, South Sudan, Sudan, Tanzania, and Uganda were the key terms used during literature searching. The Boolean operators, AND, and OR were used to combine the search terms. The reference lists of the selected articles were also screened to increase the chance of obtaining more articles. All articles that fulfilled the selection criteria were used for analysis in the review.

### 2.3 Eligibility criteria

This study included studies that specifically reported carbapenem-resistant *E. coli* originated from human, animal, food and environment in East African countries. Studies with adequate data for carbapenem-resistant *E. coli* done in these 13 East Africa countries, full-length published original research articles, written in the English language and published from September 1, 2015, to September 30, 2025 (ten years study) were included. Studies that did not report carbapenem resistant isolates, review articles, cases studies, case reports, studies with incomplete data (e.g. with only abstract), published before September 1, 2015, and after September 30, 2025, and reported from other countries were excluded from the analysis.

### 2.4 Study quality assessment

Each study was assessed and examined for the determination of eligible studies with two independent reviewers (TZ and DN) using predetermined inclusion criteria.

### 2.5 Screening and data extraction

Preliminary screening of the collected studies was done based on titles and abstracts. Two reviewers determined the eligibility of each article for inclusion and to determine their relevance. Any disagreement between the reviewers was settled through a mutual discussion to reach a final decision.

During the extraction phase, each study was critically examined by the two reviewers (TZ and BM). Discrepancies in the data extracted by the reviewers were addressed and resolved by a third reviewer's intervention. The data of selected articles were then coded, and extracted in a table in Microsoft Excel, consisting of the author's name, study participants, sample size, sample type, isolate number, carbapenem resistance frequency, carbapenem resistance prevalence, confirmatory method used, method used in gene detection, detected genes, and type of carbapenemase.

### 2.6 Evaluation of bias

All studies included in the present review were conducted using cross-sectional study design. Biases in individual studies were assessed based on the Newcastle Ottawa Scale [17] and three assessment criteria namely selection, comparability, and outcome criteria were used. Each study was rated out of four stars for selection, two stars for comparability, and three stars for the outcome of interest. The study with six stars or above were classified as having low-risk biases, those with four to five stars as having moderate-risk bias, and those with below four stars as having high-risk biases. In the evaluation of bias analysis using the assessment criteria, two individuals (T.Z and B.M) independently assessed the individual studies, and disagreement in the assigned number of stars was discussed and reviewed (Supplement Table S1). All the studies included in this review were scored 6 and were classified as having low-risk biases.

### 2.7 Analysis

The meta-analysis was performed using MetaProp codes in the meta (version 8.2-1) and Metafor (version 4.8-0) packages of R (version 4.5.2), as implemented in R Studio (version 2025.09.2-418). The pooled prevalence of resistance to any carbapenems, 95% confidence interval (CI) were calculated using the Restricted Maximum Likelihood (REML) method for the random-effects model. REML was used to estimate between-study variance (τ^2^) in the random-effects model. Statistical heterogeneity between studies was evaluated using the tau-squared (τ^2^) test and I-squared statistic (I^2^). [18] In quantifying heterogeneity (I^2^), the thresholds of 25%, 50%, and 75% were used to indicate low, medium, and high heterogeneity, respectively. [18] Moreover, heterogeneity was assessed with subgroup analysis. Publication bias was calculated by evaluating a funnel plot, which was tested for its significance with Egger's test.

## 3 Results

A total of 4336 published articles were found during our search at the different databases. After careful identification, screening and eligibility assessment (Fig. 1), a total of 29 articles were included in the final analysis. In the first identification, 4232 articles were excluded due to duplications and irrelevant articles. Systematic Reviews, meta-analysis and articles that were not done in the 13 East Africa countries, and articles that were not retrieved and not fully accessed were excluded from the review. An in-depth review of the articles also excluded 4 articles due to lack of relevant data for addressing the study objectives. Finally, 29 articles were included for the analysis of the present study.

### 3.1 General studies characteristics

The present review includes studies done from September 1, 2015, to September 30, 2025 (ten years study). Five of them (17.2%) were done between 2015 and 2020, whereas the majority (82.8%) were done between 2021 and 2025 (Table 1). Majority of the studies, 14 (48.3%) were done in Ethiopia, 7 (24.1%) in Uganda, 4 (13.8%) in Sudan, 2 (6.9%) in Tanzania, and each (3.4%), in Djibouti and Kenya. No relevant studies were found in the other countries. All studies were cross sectional study. Twenty-two (75.9%) were hospital based (including one hospital environment), the remaining 4 (13.8%) and 3 (10.3%) were community based and environmental based setting, respectively (Supplement file).

**Table 1:** Summary of 29 studies reporting the prevalence of carbapenem resistance bacteria in different parts of East Africa countries from 2015 to 2025.

Study ID	Publication year	Study country	Category	Sample size	Isolate number	CarbR isolate	CarbR Prevalence	Pooled prevalence(95%CI)
Alelign_2023(19)	2023	Ethiopia	Food	100	39	3	7.70%	7.7% (0.16, 2.09)
Tilahun_2024 [19]	2024	Ethiopia	Human	810	79	21	26.90%	26.6% (1.73, 3.77)
Befikadu_2024 [20]	2024	Ethiopia	Human	344	292	11	3.80%	3.8% (0.19, 0.66)
Ragueh_2023 [21]	2023	Djibouti	Human	235	131	13	10%	9.9% (0.54, 1.64)
Tekele_2021 [22]	2021	Ethiopia	Human	312	226	3	1.30%	1.3% (0.03, 0.38)
Dirar_2020 [23]	2020	Sudan	Human	168	81	4	4.90%	4.9% (0.14, 1.22)
Odoko_2024 [24]	2024	Ethiopia	Human	422	29	4	13.80%	13.8% (0.39, 3.17)
Macha_2025 [25]	2025	Tanzania	Human	326	219	8	3.70%	3.7% (0.16, 0.71)
Tilahun_2022 [26]	2022	Ethiopia	Human	384	80	9	11.20%	11.2% (0.53, 2.03)
Gebremedhin_2023 [27]	2023	Ethiopia	Human	67	46	2	4.30%	4.3% (0.05, 1.48)
Mekonnen_2023 [26]	2023	Ethiopia	Human	384	307	37	12.40%	12.4% (0.86, 1.62)
Worku_2022 [28]	2022	Ethiopia	Human	384	219	1	0.50%	0.5% (0.00, 0.25)
Sebre_2021 [29]	2021	Ethiopia	Environment	103	64	12	18.80%	18.8% (1.01, 3.05)
Kiros_2023 [30]	2023	Ethiopia	Human	383	89	31	34.8%	34.8% (2.50, 4.57)
Mutuma_2023 [31]	2023	Kenyan	Human	267	267	6	2.20%	2.2% (0.08, 0.48)
Shibabaw_2023 (34)	2023	Ethiopia	Human	384	74	8	10.80%	10.8% (0.48, 2.02)
Amare_2022 (35)	2022	Ethiopia	Human	290	245	7	2.90%	2.9% (0.12, 0.58)
Tuhamize_2023 (36)	2023	Uganda	Human	96	96	2	2.10%	2.1% (0.03, 0.73)
Tuhamize_2023 (36)	2023	Uganda	Animal	96	96	4	4.20%	4.2% (0.11, 1.03)
Tuhamize_2024 (37)	2024	Uganda	Human	2371	213	43	20.20%	20. 2% (1.50, 2.62)
Okoche_2015 (38)	2015	Uganda	Human	196	84	9	10.70%	10.7% (0.50, 1.94)
Kimera_2021 (39)	2021	Tanzania	Environment	219	63	5	8%	7.9% (0.26, 1.76)
Mahmoud_2020 (40)	2020	Sudan	Environment	150	45	13	29%	28.9% (1.64, 4.43)
Ampaire_2015 (41)	2015	Uganda	Human	658	99	26	26.30%	26.3% (1.79, 3.61)
Elbadawi_2021 (42)	2021	Sudan	Human	206	28	12	42.80%	42.9% (2.45, 6.28)
Bagaya_2023 (43)	2023	Uganda	Environment	104	20	10	50%	50% (2.72, 7.26)
Eshetie_2015 (44)	2015	Ethiopia	Human	442	112	2	1.80%	1.8% (0.02, 0.63)
Hamid_2021 (45)	2021	Sudan	Human	1062	144	40	27.80%	27.8% (2.06, 3.58)
Ssekatawa_2021 (46)	2021	Uganda	Human	618	421	96	22.80%	22.8 (1.89, 2.71)

The source of the samples was from human (23, 79.3%), environment (4, 13.8%), and from food and animal, each 1 (3.4%) (Table 1). The total sample size of the present study was 11581. Total *E. coli* isolates were 3908 with 442 carbapenem-resistant *E. coli* strains. Antimicrobial resistance genes were detected in 15 (51.7%) of the studies. The resistance genes include KPC, OXA-48, NDM-1&5, OXA-181, OXA-181, VIM, IMP, GES, IM, and SPM (Table 1).

### 3.2 Prevalence of carbapenem-resistant *E. coli*

**Fig. 2:**
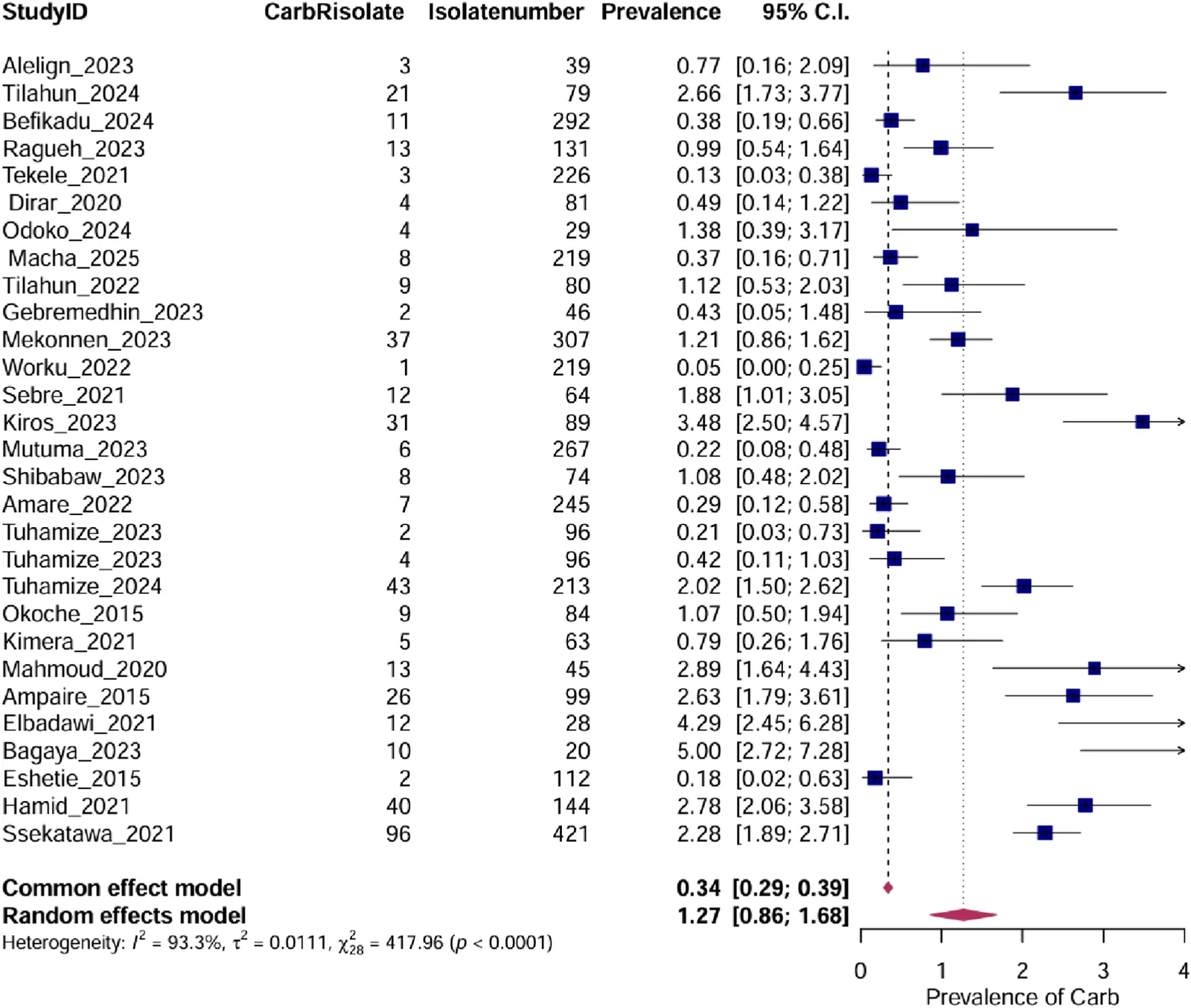
Forest plot of the pooled prevalence of carbapenem-resistance *E. coli* in East Africa countries from 2015 to 2025.

**Fig. 3:**
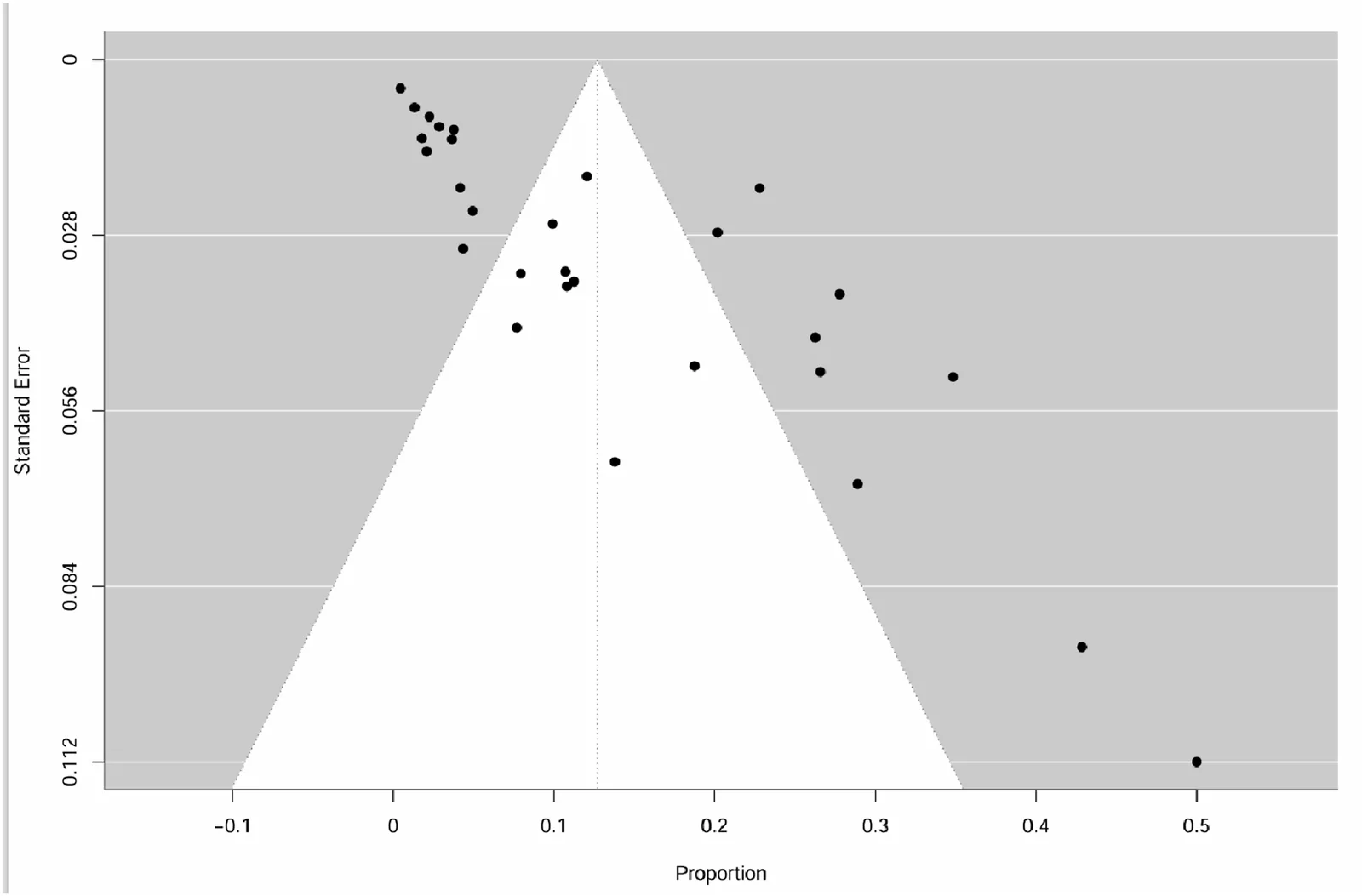
Funnel plot with 95% confidence limits of the prevalence of carbapenem-resistance *E. coli* in East Africa countries from 2015 to 2025.

The pooled prevalence was determined using pscale = 10 (Fig. 2). The pooled prevalence of carbapenem-resistant *E. coli* in the present study was 12.7% (95% CI: 8.6, 16.8) (Fig. 2). The low prevalence was 0.5% from Ethiopia [32] and high prevalence was 50% from Uganda. [19] The tau-squared (τ2) at 0.0111, estimated the variability in the true effect sizes across the included studies, the heterogeneity was extremely high (I^2^ = 93.3%) indicating variability in effect sizes across studies. These were further supported by the Cochran's Q-statistic (Q = 417, p < 0.0001) showed the observed heterogeneity in study outcomes differ statistically rather than by random sampling error. Since the value of I^2^ is greater than 75%, it indicates that there is high heterogeneity among the studies. The funnel plot (Fig. 3) shows the distribution of the studies and that there is clear evidence of heterogeneity and funnel plot asymmetry. In addition, meta-regression analysis identified that the reported frequency of carbapenem resistant in the studies was found a significant contributor to the observed heterogeneity (p < 0.001). The Egger's test showed that there was statistically significant publication bias in estimating the prevalence of carbapenem-resistant *E. coli* (Test for Funnel Plot Asymmetry: z = 7.2618, p < 0.0001). The trim and fill plot was used to adjust the effect of publication bias on the estimation of the pooled prevalence.

### 3.3 Subgroup analysis

The pooled prevalence of Carbapenem-resistant *E. coli* was higher between 2015 and 2020 compared to the studies conducted between 2021 and 2025 (Table 2, Supplement figure Fig. S1–4). The publication years were classified into two, with a five-year period, to capture potential epidemiological changes in carbapenem resistant of *E. coli* in the region. This is due to that the resistance pattern, the healthcare intervention practices, and other relevant factors may change over time. The high prevalence was seen in Sudan (24.7%) (Table 2), followed by Uganda (17.4%) and Ethiopia (9.9%). Environment was found the predominant interface with pooled prevalence of Carbapenem-resistant *E. coli* (24.2%) followed by human (11.9%). The pooled prevalence of this Carbapenem-resistant *E. coli* was high (15.8%) *in* various clinical samples (>1 type) next to environmental samples (24.2%).

**Table 2:** Subgroup estimate pooled prevalence of Carbapenem resistant *E. coli* by different characteristics in East Africa countries from 2015 to 2025.

Variables	Characteristics	Included studies	Sample size	Isolate number	Carba resistance isolate	Pooled prevalence 95% CI
Publication year	2015-2020	5	1614	421	54	13.5 (0.30, 2.40)
	2021-2025	24	10351	3561	396	12.6 (0.81, 1.71)
Country	Ethiopia	14	5193	1975	159	9.9 (0.48, 1.49)
	Uganda	7	4139	1029	190	17.4 (0.74, 2.74)
	Sudan	4	1586	298	69	24.7 (0.92, 4.02)
	Tanzania	2	545	282	13	0.5 (0.01, 0.08)
	Kenya	1	267	267	6	2.2% (0.08, 0.48)
	Djibouti	1	235	131	13	1.0 (0.05, 0.16)
Study participant category	Human	23	11193	3655	403	11.9 (0.74, 1.63)
	Environment	4	576	192	40	24.2 (0.82, 4.03)
	Animal	1	96	96	4	0.4 (0.01, 0.10)
	Food	1	100	39	3	0.8 (0.02, 0.21)
Types of samples	Various clinical samples (>1 type)	11	5359	1660	248	15.8 (0.88, 2.27)
	Stool samples	8	2244	1611	99	7.1 (0.02, 1.39)
	Urine samples	3	2880	371	47	8.6 (0.00, 1.99)
	Environmental samples	4	576	192	40	24.2 (0.82, 4.03)
	Surgical samples	2	806	109	13	1.2 (0.06, 0.18)
	Food sample	1	100	39	3	0.8 (0.02, 0.21)

## 4 Discussion

This systematic review and meta-analysis study was conducted by exploring published articles in East African countries from different databases. The study is a one-health approach including different interfaces. *E.coli* is an excellent indicator organism to assess the dynamics of AMR in a One-health perspective. [13] The extent of the carbapenem-resistant problems seen in this organism could reflect for other bacteria. The pooled prevalence of carbapenem-resistant *E. coli* with a one-health approach in East Africa, and subgroup analysis for year of publication, countries, different interfaces, and types of samples has been determined in this study.

The pooled prevalence of carbapenem-resistant *E. coli* with a one health approach in East Africa was 12.7%. A previous systematic review study done in 2018 by Ssekatawa *et al* in East Africa reported 12% carbapenem-resistant *E. coli* [20] which is consistent with the present study. Other similar studies reported carbapenem-resistant *E. coli* include 14.2% in Nigeria, [21] 5.0% in Iran, [22] and 3.9% in Saudi Arabia. [23] The high prevalence in the present study compared to studies done in Iran and Saudi Arabia may be due to difference in contributing factors present in low- and high-income countries. For example, in low-income countries the contributing factors are high due to weak laboratory capacity, poor health systems governance, lack of health information systems, and limited resources. [24] Overcoming challenges through providing service or infrastructure that allow timely detection for action could reduce the problem.

The studies included in this review adopted a One Health approach and showed substantially variations in their characteristics. Although patients accounted for 72.4% of the study population, the remaining studies involved diverse sources, including food items, environmental samples, food handlers, farmers, and animals. Furthermore, the studies were conducted across different countries, with nearly half (48.3%) originating from Ethiopia. Considerable variation in study settings was also observed, 75.9% were hospital-based, while the remainder were conducted in community or environmental settings. Similarly, the sample sources differed markedly across studies included in the review. This diversity in study populations, geographical distribution, study settings, and sample sources reflect the One Health nature of the included studies and is a likely contributed to the substantial heterogeneity observed in the meta-analysis. Furthermore, subgroup analyses were conducted to explore the sources of heterogeneity. The findings demonstrated substantial variation in heterogeneity across subgroups. By country, heterogeneity ranged from low in Tanzania (I^2^ = 28%) to very high in Uganda (I^2^ = 95%). Similarly, studies involved human participants showed heterogeneity (I^2^ = 94.1%) by study population, with studies involving humans showing high heterogeneity. Differences were also observed according to sample source, various sample sources exhibited high heterogeneity (I^2^ = 95%), whereas studies based solely on urine samples showed no heterogeneity (I^2^ = 0%). These findings suggest that geographical differences, variations in study populations, and differences in sample sources were important contributors to the overall heterogeneity observed in the present meta-analysis.

The pooled prevalence of carbapenem *E. coli* was 24.2% in the environment, 11.9% in humans, 7.7% in food, and 4.2% in animals. Different studies reported prevalence of carbapenem-resistant bacteria from different interfaces. [25,26] Dossouvi et al. [26] reported 19.1% of the pooled prevalence of carbapenem-resistant bacteria in Africa. Developing countries are faced with different challenges, [27] and the disposal of human and animal wastes close to human residences and lack of access to clean water are also documented in these countries. [27] A study done in Africa found carbapenem-resistant bacteria in 59.1% of animals, 48.2% of the environment, and 19% of food, [26] reflecting that the different interfaces are contributing to its occurrence in the region. The present study finding could also reflect the gaps in collaborative action across human, animal, and environmental sectors to mitigate the challenges with the One Health initiative.

In this study, the prevalence of carbapenem-resistant *E. coli* was reduced for the year from 2021 to 2025 compared to the year from 2015 to 2020. The Public Health Institute of Amhara Region reported trends in AMR (2016 - 2021) and found that the overall trend of AMR has been rising from year to year, reaching a peak in 2019 then after declined. [28] The trends for the prevalence of Carbapenem-resistant bacteria varied; in most case increasing time to time. [29] In the present study, the difference was not statistically significant and could reflect possible variation due to the study design, study area, and the target bacteria. The finding could indicate an urgent action through advancement of interventions through time.

High prevalence of carbapenem-resistant *E. coli* was in Sudan (24.7%) followed by Uganda (17.4%) and Ethiopia (9.9%) compared to other countries. Among the significant factor that contributes to the emergence of AMR in East Africa countries include prolonged use, overuse, or misuse of antimicrobial drugs in human and animal populations. [30] The problem would be more multiplied by the conflict and instability in East Africa countries including Sudan and Ethiopia, and this could contribute the high prevalence of carbapenem-resistant *E. coli*. Integrated not only with one health approach at different interfaces but also at regional level beyond health policy demands the problem.

Different resistance genes including serine β-lactamases (Calss A: KPC and GES), metallo-β-lactamases (Calss B: NDM-1&5, VIM, IMP and SPM), and oxacillinase (class C: OXA-48, OXA-181, and OXA-181) were found in the present study. Now a days, gene-encoding carbapenemase such as blaKPC reported in Environment (24.8%), human (7.6%), and animal (1.3%). [25] One important mechanism for occurrence of carbapenem-resistant bacteria is acquiring gene-encoding carbapenemase. Due to the lack of routine testing which resulted in the low data volume in Africa, [31] true prevalence may not be represented with the present study. However, in the present study, different carbapenem-resistant encoding genes were reported in 51.7% of the studies. The gene-encoding carbapenemase could be potential threats to the region due to their rapid spread, and lack of routine detection methods in clinical laboratories. National or regional surveillance, rapid detection methods, monitoring programs, and further research could help to mitigate the problem.

The present study adopted a One Health approach providing a comprehensive assessment of Carbapenem-resistant *E. coli* across multiple sectors, including humans, animals, food, and the environment. This broad perspective highlights the multisectoral nature of AMR and underscores the need for coordinated One Health collaboration to address this growing public health threat. However, the review has several limitations. The literature search was restricted to three electronic databases (PubMed, ScienceDirect, and Google Scholar), and only English language published studies with cross-sectional study design were included. As a result, relevant studies indexed in other databases or published in other languages may have been missed, potentially introducing publication and language bias.

Carbapenem-resistant *E. coli* was found prevalent in East Africa among different interfaces so that the finding could reflect Carbapenem-resistant bacteria as a threat in the region. Multifaceted strategies or interventions require to mitigate the problem. Some of the intervention could include effective implementation of one-health approach, improvement of routine diagnostic capacity, and regional integration beyond health policy.

## Authors’ contributions

TZ was the principal investigator who contributed to origin, the idea and design of the study. TZ, BM and DN collected, entered, analysed, interpreted the data, prepared the manuscript, contributed to data analysis, interpretation and drafted the manuscript. All authors read and approved the final manuscript.

## Ethical statement

This study is a systematic review and meta-analysis based on previously published data and does not involve human participants or the collection of primary data. Therefore, ethical approval is not required.

## Availability of data and materials

All information is clearly presented in the main manuscript.

## Funding

Since this study was systematic review ,there was no funding or sponsoring organization for this paper.

## Declaration of competing interest

The manuscript also meets its policies. All authors read the final manuscript and approved the manuscript submission. The manuscript has not been published and is not under consideration for publication elsewhere. We have no conflict of interest to disclose.
