# Supplementary material for: Carbapenem-resistant E. coli with one-health approach in East Africa: Systematic review and meta-analysis
Source: Public Health Pract (Oxf). 2026 Aug 22;12:100843. doi: 10.1016/j.puhip.2026.100843 (PMC13544070; doi:10.1016/j.puhip.2026.100843)
Supplement: Supplementary file 1 [file mmc1.docx]

**Table S1. Evaluation of bias** **in individual studies using the Newcastle-Ottawa Scale (NOS) for assessing non-randomized studies**

| **Reference** | **Selection** | | | | **Comparability** | **Outcome** | | |  |  |
| --- | --- | --- | --- | --- | --- | --- | --- | --- | --- | --- |
|  | Representative of the study cases | Selection of Comparison Samples (if applicable) | Ascertainment of Resistant Bacteria | Demonstration Not Prevalent at Baseline | Control for Confounding Variables | Assessment of Resistant Bacteria | Was Sampling and Follow-Up Long Enough for Reliable Results | Adequacy of Sampling and Data Reporting | Total Score | Quality |
| Alelign et al, 2023 | * | - | * | * | - | * | * | * | 6 | Good |
| Tilahun et al, 2024 | * | - | * | * | - | * | * | * | 6 | Good |
| Befikadu et al, 2024 | * | - | * | * | - | * | * | * | 6 | Good |
| Ragueh et al, 2023 | * | - | * | * | - | * | * | * | 6 | Good |
| Tekele et al, 2021 | * | - | * | * | - | * | * | * | 6 | Good |
| Dirar et al, 2020 | * | - | * | * | - | * | * | * | 6 | Good |
| Odoko et al, 2024 | * | - | * | * | - | * | * | * | 6 | Good |
| Macha et al, 2025 | * | - | - | * | - | * | * | * | 6 | Good |
| Tilahun et al, 2022 | * | - | * | * | - | * | * | * | 6 | Good |
| Gebremedhin et al, 2023 | * | - | * | * | - | * | * | * | 6 | Good |
| Mekonnen et al, 2023 | * | - | * | * | - | * | * | * | 6 | Good |
| Worku et al, 2022 | * | - | * | * | - | * | * | * | 6 | Good |
| Sebre et al, 2021 | * | - | * | * | - | * | * | * | 6 | Good |
| Kiros et al, 2023 | * | - | * | * | - | * | * | * | 6 | Good |
| Mutuma et al, 2023 | * | - | * | * | - | * | * | * | 6 | Good |
| Shibabaw et al, 2023 | * | - | * | * | - | * | * | * | 6 | Good |
| Amare et al, 2022 | * | - | * | * | - | * | * | * | 6 | Good |
| Tuhamize et al, 2023 | * | - | * | * | - | * | * | * | 6 | Good |
| Tuhamize et al, 2023 | * | - | * | * | - | * | * | * | 6 | Good |
| Tuhamize et al, 2024 | * | - | * | * | - | * | * | * | 6 | Good |
| Okoche et al, 2015 | * | - | * | * | - | * | * | * | 6 | Good |
| Kimera et al, 2021 | * | - | * | * | - | * | * | * | 6 | Good |
| Mahmoud et al, 2020 | * | - | * | * | - | * | * | * | 6 | Good |
| Ampaire et al, 2015 | * | - | * | * | - | * | * | * | 6 | Good |
| Elbadawi et al, 2021 | * | - | * | * | - | * | * | * | 6 | Good |
| Bagaya et al, 2023 | * | - | * | * | - | * | * | * | 6 | Good |
| Eshetie et al, 2015 | * | - | * | * | - | * | * | * | 6 | Good |
| Hamid et al, 2021 | * | - | * | * | - | * | * | * | 6 | Good |
| Ssekatawa et al, 2021 | * | - | * | * | - | * | * | * | 6 | Good |

**Note**: ‘*’ indicate that the article meets the criteria mentioned above.
