# Supplementary material for: Carbapenem-resistant E. coli with one-health approach in East Africa: Systematic review and meta-analysis
Source: Public Health Pract (Oxf). 2026 Aug 22;12:100843. doi: 10.1016/j.puhip.2026.100843 (PMC13544070; doi:10.1016/j.puhip.2026.100843)
Supplement: Supplementary file 2 [file mmc3.docx]

**Fig a. From 2015-2020**

**Fig b. From 2021 to 2025**

**Fig S1. Pooled prevalence of carbapenem-resitant *E. coli* by publication years**

**Fig S2. Pooled prevalence of carbapenem-resitant *E. coli* by countries**

**Fig S3. Pooled prevalence by different interfaces**

**Fig S4. Pooled prevalence by samples**
